# Multimodal fluorescence-optoacoustic *in vivo* imaging of the near-infrared calcium ion indicator NIR-GECO2G

**DOI:** 10.1016/j.pacs.2024.100671

**Published:** 2024-11-28

**Authors:** Sarah F. Shaykevich, Justin P. Little, Yong Qian, Marie-Eve Paquet, Robert E. Campbell, Daniel Razansky, Shy Shoham

**Affiliations:** a NYU Langone Health, Tech4Health and Neuroscience Institutes, and Department of Ophthalmology, New York City, USA; b Massachusetts Institute of Technology, McGovern Institute for Brain Research, Cambridge, MA, USA; c The University of Tokyo, Department of Chemistry, Tokyo, Japan; d CERVO Brain Research Centre, Québec, Canada; e Université Laval, Department of Biochemistry, Microbiology and Bioinformatics, Québec, Canada; f University of Zurich, Institute of Pharmacology and Toxicology and Institute for Biomedical Engineering, Faculty of Medicine, Zurich, Switzerland; g ETH Zurich, Institute for Biomedical Engineering, Department of Information Technologies and Electrical Engineering, Zurich, Switzerland

**Keywords:** Calcium indicators, In vivo imaging, Brain, Near-infrared, Fluorescence, Biliverdin, Optoacoustic tomography

## Abstract

Measuring whole-brain distributed functional activity is an important unmet need in neuroscience, requiring high temporal resolution and cellular specificity across large volumes. Functional optoacoustic neuro-tomography (FONT) with genetically encoded calcium ion indicators is a promising approach towards this goal. However, it has not yet been applied in the near-infrared (NIR) range that provides deep penetration and low vascular background optimal for *in vivo* neuroimaging. Here, we study the noninvasive multimodal fluorescence and optoacoustic imaging performance of state-of-the-art NIR calcium ion indicator NIR-GECO2G in the mouse brain. We observe robust *in vivo* signals with widefield fluorescence, and for the first time, with FONT. We also show that in both modalities, the NIR-GECO2G signal improves more than twofold in the biliverdin-enriched *Blvra*^*-/-*^ mouse line compared to wild type. Our findings demonstrate the potential of multimodal fluorescence and optoacoustic NIR imaging, opening new possibilities for whole-brain real-time functional imaging in rodents.

## 1 Introduction

Genetically encoded calcium ion (Ca^2+^) indicators (GECIs) are ubiquitous in microscopic neuroimaging applications because they provide a direct functional fluorescence signal that enables the targeted recording of the activity of specific neuronal subpopulations with a relatively high temporal resolution [1]. As a result, they have become indispensable tools in systems neuroscience in conjunction with various microscopy and photometry techniques. However, most optical methods are not well suited to imaging large areas at depth, which limits the utility of fluorescence readouts of neural activity. This fundamental limitation can be overcome with hybrid optoacoustic (OA) methods such as functional optoacoustic neuro-tomography (FONT) [2], [3]. FONT offers spectroscopic imaging capability for sensitive differentiation between the intrinsic tissue absorbers such as hemoglobin and extrinsic markers of neural activity [4]. Hence, it is particularly well-poised to enable the imaging of GECIs throughout the entire rodent brain. Since the technique has also been shown to enable ultrafast volumetric image acquisitions at up to kilohertz-range frame rates [5] at (scalable) spatial resolutions down to 35 µm [6], the combination of GECIs and FONT opens exciting possibilities for examining complex neural systems.

While recent work has shown that GECIs can be compatible with FONT, these demonstrations have thus far been limited to GCaMP-family variants [3], [4], [7], [8]. This family of bright GFP-based GECIs has been optimized for fluorescence imaging and has found widespread use in functional neuroimaging. However, its optical absorption peak in the visible band (∼495 nm) [9], [10] limits the depth of microscopy-based visualization into the mammalian brain due to the low penetrability of blue light [4]. To truly take advantage of whole-brain imaging capabilities afforded by FONT, GECIs operating in wavelength ranges more suitable for deep tissue imaging must be explored. The 650–950 nm NIR I range is ideal for tissue penetration due to lower absorption and scattering in tissue, but GECIs in this range have not yet been used for OA detection, despite the promise of FONT imaging at centimeter-scale depths [11].

Notably, recent years have seen the introduction of several promising NIR GECIs, including multiple variants of the NIR-GECO [12], [13], [14] and iGECI [15], [16] families. In addition to improved imaging depth and lower background signal under both fluorescence and FONT modalities, NIR indicators also have the potential for multiplexed use in combination with other visible indicators and optogenetic activators due to the minimization of spectral overlap [17]. The first and furthest red-shifted type of NIR GECI is the NIR-GECO family of indicators, with the NIR-GECO1, NIR-GECO2, and NIR-GECO2G variants based on the fluorescent protein mIFP. NIR-GECOs have an absorption peak at ∼678 nm and emission peak at ∼704 nm [12], positioning them within an ideal window for light penetration in tissue [11]. Thus far, the NIR-GECO2G variant is one of the most promising NIR GECIs for *in vivo* and FONT imaging applications due to its biocompatibility and sensitivity to Ca^2+^ [13].

While being an exciting new development, NIR GECIs still suffer from deficits in performance when compared to state-of-the-art visible GECIs, including their photostability, kinetics, and brightness characteristics. One limiting factor of the NIR fluorescent proteins used in existing NIR GECIs is their dependence on endogenous biliverdin IXα, a heme catabolism product, as a chromophore. The brightness of biliverdin-based fluorescent proteins relies on the biliverdin binding efficiency of the protein as well as the availability of biliverdin. Introduction of exogenous biliverdin can increase fluorescence of biliverdin-based fluorescence proteins, but low membrane permeability limits the extent of this approach. A new method of effectively increasing biliverdin concentration was developed using *biliverdin reductase-a* knockout mice (*Blvra*^*-/-*^), which lack the biliverdin reductase-A (BLVRA) enzyme that is responsible for the reduction of biliverdin to bilirubin in adult mammalian tissues[18]. The *Blvra*^*-/-*^ mouse line has been shown to increase NIR-GECO1 fluorescence in mouse embryonic fibroblast cells[2].

In this work we provide the first characterization of NIR-GECO2G for *in vivo* multimodal fluorescence-FONT imaging in mice, towards the goal of optimizing imaging fidelity. We show that NIR-GECO2G can be used for *in vivo* mouse functional neuroimaging experiments and that its performance in mice is improved over its predecessor NIR-GECO1. We then use spectroscopic FONT to demonstrate for the first time the robust observation of this NIR GECI’s OA signature *in vivo*. As a potential strategy for optimizing signal strength, we show that in *Blvra*^*-/-*^ mice, both NIR-GECO2G’s fluorescence and OA signal strengths are increased by a factor of ∼2.5 over wild-type mice. Additionally, we investigate the susceptibility of NIR-GECO2G to photobleaching and potential strategies for extended imaging time. We finally demonstrate fully hybrid (concurrent) fluorescence and FONT *in vivo* imaging of NIR-GECO2G, setting the stage for further advances and applications of volumetric brain-wide multimodal functional rodent brain imaging.

## 2 Methods

### 2.1 Imaging systems

**Fig. 1:**
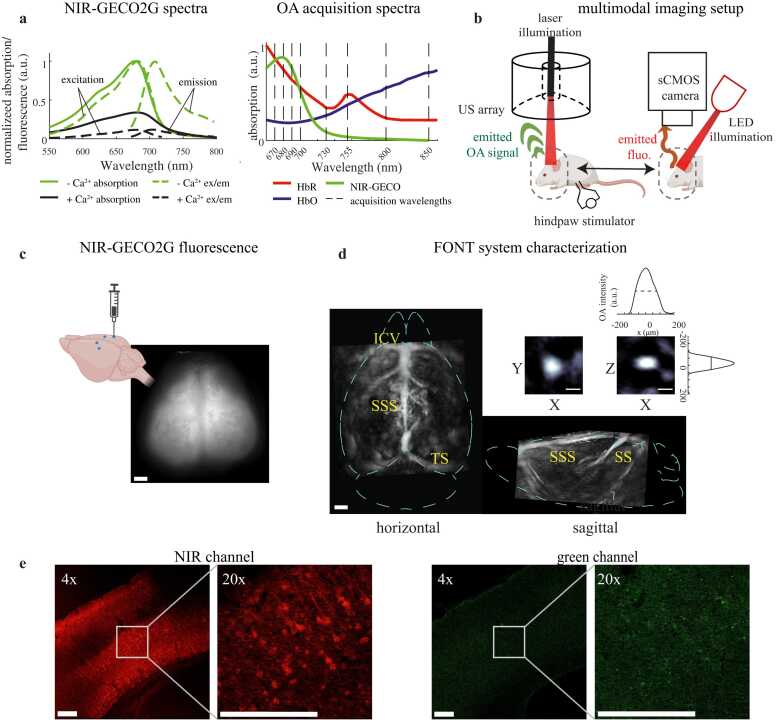
Setup for imaging of NIR-GECO2G in the mouse brain. (a) Light spectra relevant to NIR-GECO2G. Left: NIR-GECO2G excitation and emission curves are shown for Ca^2+^-saturated NIR-GECO2G (black dashed lines) and Ca^2+^-free NIR-GECO2G (green dashed lines), as well as absorption spectra of NIR-GECO2G with (solid black line) and without (solid green line) the presence of Ca^2+^ (data from ref. [13]). Right: absorption spectra of expected tissue components NIR-GECO2G (green), deoxyhemoglobin (HbR, red), and oxyhemoglobin (HbO, blue) and wavelengths used for imaging (gray dashed lines), each scaled to equivalent area under the curve. (b) Diagram of experimental setups. (US = ultrasound). (c) Representative *in vivo* NIR-GECO2G expression in a 6-week-old mouse brain on the widefield imaging setup. Scale bar = 1 mm. The brain illustration at the upper left shows approximate injection sites (d) Maximal intensity projections of OA signal collected from a 6-week-old mouse brain at 800 nm (SSS = superior sagittal sinus, TS = transverse sinus, ICV = inferior cerebral vein, SS = straight sinus). Scale bar = 1 mm. Inset: Maximal intensity projections and intensity profiles for a single phantom sphere used for resolution characterization. Scale bar = 100 μm. (e) Expression pattern of NIR-GECO2G across cortical layers. In the NIR channel, neurons labeled with NIR-GECO2G are seen in cortical layers 2/3, 5, and 6. No significant fluorescence above autofluorescence is seen in the green channel. Scale bars = 200 μm.

We performed multimodal fluorescence-FONT imaging, either with sequential application of widefield fluorescence imaging and FONT (Fig. 1) or simultaneously using a fiberscope-based hybrid imaging system [19], [20] (Fig. 5(a)).

For standalone widefield imaging, illumination is provided by a 660 nm light-emitting diode (LED, SOLIS-660C, Thorlabs, USA). Emission light is collected through a macro lens (Zoom 7000, Navitar, USA) with 6x magnification and a focal length of 18–108 mm, at a working distance of ∼170 mm, and filtered through a 775±70 nm bandpass filter (same as used in fiberscope experiments below) to approximate the function of a longpass filter. Images are captured by an sCMOS camera (FL-20BW, Tucsen, China) at 6.67 Hz with an exposure time of 120 ms.

The FONT system used in this study includes a custom ultrasound transducer array (Imasonic, France) with a central frequency of 7.2 MHz and a −6 dB detection bandwidth of 4.6 MHz. The array consists of 512 piezoelectric ultrasound transducers arranged in rings around a hemispherical surface with a radius of 4 cm and acceptance angle of 140°. An 8 mm diameter bore in the center of the array allows a fiber bundle (CeramOptec, Germany) to be inserted directly above the array to illuminate the subject below. The fiber bundle is coupled to an optical parametric oscillator (OPO) laser (SpitLight EVO II, InnoLas Laser, Germany) that emits 5–8 nanosecond pulses at up to 200 Hz and and provides rapid wavelength tuning capability on a per-pulse basis in the 670–980 nm spectral window. The optoacoustically-generated signals are detected by the 512 elements of the transducer array and simultaneously digitized by a custom data acquisition system at 40 MS/s (Falkenstein Mikrosysteme, Germany). Data acquisition is triggered by a TTL signal sent from the OPO laser upon each emitted light pulse. The digitized signals are sent to a computer equipped with a GPU (Radeon Pro WX 9100, Advanced Micro Devices, USA) for real-time reconstruction [21] and are also saved for later offline processing.

We evaluated the system’s performance characteristics both using phantoms and *in vivo* (Fig. 1(d)). Optoacoustic signal is clearly detected down to at least 5 mm depths in the brain. Imaging resolution was measured using ∼50 μm diameter microspheres suspended in 2 % agar (Cospheric, USA), resulting in a lateral resolution of ∼156 ± 12 μm and axial resolution of ∼95 ± 3 μm (± standard deviation of the mean for 10 independent measurements). These results agree with the predicted approximations for lateral resolution ($R_{L}=\frac{v_{s}}{\sin(\alpha )∙f_{0}}\;\approx \;$157 μm for the array’s acceptance angle α, central frequency $f_{0}$, and speed of sound $v_{s}$=1500 m/s in tissue) [22] and axial resolution ($R_{A}=0.6\lambda_{c}\approx$ 95 μm for the wavelength at the upper cutoff frequency of our array, $\lambda_{c}$) [23], [24], [25].

### 2.2 Constructs, vectors and animal preparation

The DNA sequence encoding NIR-GECO2G was cloned into the pAAV plasmid with the neuron specific hSynapsin promoter for recombinant AAV production. Briefly, AAVs were generated in HEK293T17 cells by triple transfection with pXX680 helper plasmid plus plasmids encoding rep/cap and NIR-GECO2G. Forty-eight hours post transfection, cells were harvested and viral particles released by freeze-thaw cycles on dry-ice/ethanol and free DNA digested with benzonase. AAV particles were purified by a discontinuous gradient of iodixanol and ultracentrifugation. The viral preparation was then washed and concentrated through a 100 kDa amicon filter. Titration was performed by TaqMan digital droplet-PCR using primers specific for inverted terminal repeats of AAV2. Three- to four-week-old B6N-Tyrc-Brd/BrdCrCrl mice (Charles River Laboratories, USA) and *Blvra*^*-/-*^ mice (Laboratory Animal Resource Bank at National Institutes of Biomedical Innovation, Health and Nutrition, Japan) for the FONT and multimodal experiments, and three- to four-week old wild-type (B6N-Tyrc-Brd/BrdCrCrl or C57/BL6) and *Blvra*^*-/-*^ mice for widefield fluorescence, were injected with AAV2/9-hSyn-NIR-GECO2G at a concentration of 2.1×10^13^ genome copies/mL. The mice were anesthetized with 1.5–2 % *v/v* isoflurane throughout surgery with a flow rate of 1 L/min (3 % isoflurane for induction). A caudorostral incision was made in the center of the mouse’s scalp between the eyes and lambda. A dental drill was used to thin the skull at four injection points, then a microinjection pipette was inserted using a nanoinjector (Nanoject II, Drummond Scientific Company, USA) mounted to a micromanipulator (Sutter Instrument MP-285) at each of the four sites at 0.6 mm posterior from. bregma, ±1.2 mm lateral, and 1 mm depth (approximately sensory hindlimb cortical region in each hemisphere) or 1.2 mm posterior from bregma, ±2.5 mm lateral, and 1.2 mm depth (approximately sensory barrel field cortical region in each hemisphere, Fig. 1(c), brain diagram created with BioRender.com). 700 nL of virus were injected at each site, delivering 100 nL every minute for seven minutes and leaving the capillary inserted for 5 minutes afterwards before withdrawing. The incision was sutured following surgery.

### 2.3 Brain section preparation and imaging

A five-week-old wild-type mouse was injected with AAV2/9-hSyn-NIR-GECO2G (following the procedure outlined in Section 2.1). At 6.5 months, the mouse was anesthetized with a ketamine (100 mg/kg) and xylazine (10 mg/kg) mixture and was transcardially perfused with 10 mL 4 % paraformaldehyde solution in PBS. The brain was harvested and transferred into 30 % sucrose solution in PBS at 4°C for 24 hours and then embedded with O.C.T. compound (Fisher Healthcare). The brain was sliced into 40 μm thick coronal sections using a cryostat (Leica, model # CM3050S). The sections were mounted on slides and coverslipped using a mounting medium (Fluoromount, Diagnostic BioSystems) after drying. Images were acquired on a laser scanning confocal microscope (FLUOVIEW™ FV4000, EVIDENT, USA) with a 685 nm excitation laser and 710 – 810 nm emission detection for the red channel and 488 nm excitation laser and 500 – 600 nm emission detection for the green channel (Fig. 1(e)).

### 2.4 Electrical hindpaw stimulation

Stainless steel 30 G needles were inserted under the foot pad skin and connected to a stimulator emitting 5 V pulses (SD9, Grass Instrument Company, USA). The paw was sanitized with 70 % isopropanol prior to insertion and disinfected after experiments with bacitracin ointment.

### 2.5 In vivo widefield Ca^2+^ imaging and analysis

Following viral expression, wild-type (n = 6) and *Blvra*^*-/-*^ (n = 6) mice were anesthetized under 1–1.5 % isoflurane and their scalps were removed. Mice were then subjected to several electrical stimuli. At the end of the first experimental day, the exposed skulls were sealed with a transparent coating created by applying cyanoacrylate glue (Super Glue Ultra Liquid Control, Loctite, USA) to the skull surface, allowing it to dry for 10 minutes, and then applying a layer of clear nail polish (Out the Door Fast Drying Topcoat, INM Nails, USA). Electrical hindpaw stimulation was performed every 10 seconds for 20 trials under 0.0025 mW/mm^2^ illumination for the right hindpaw and then the left hindpaw. By random assignment, 15 trials of each experiment consisted of a single pulse of length 50 ms and 5 trials consisted of a train of 10 pulses of length 20 ms with 50 ms intervals. After the two hindpaw stimulation experiments, LED intensity was increased to 0.025 mW/mm^2^. Three separate experimental sessions were performed in order to characterize bleaching and to diminish the signal between experimental days in order to observe the consequences of decreased signal on functional readout and possible across-day signal recovery. Each bleaching session lasted 16 minutes, with reference images at 0.0025 mW/mm^2^ illumination taken after each bleaching session. In a core group of 3 wild-type and 3 *Blvra*^*-/-*^ mice, stimulation and bleaching experiments were performed every three days, on the 14, 17, and 20th day after NIR-GECO2G injection. Final stimulation experiments and reference images were taken on the 23rd day after injection. Additional wild-type mice (n = 3) were imaged at 11, 14, 17, and 20 days after injection but without bleaching sessions and with only single pulse stimuli. Additional *Blvra*^*- /-*^ mice (n = 3) were imaged at 16–17 days after injection, and only with single pulse stimuli. These two groups were imaged at a higher illumination level of 0.035 mW/mm^2^.

**Fig. 2:**
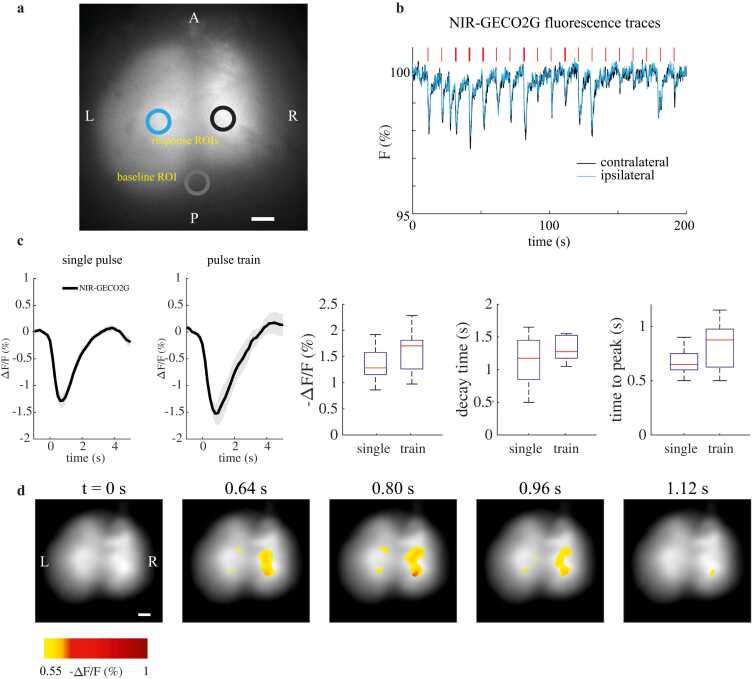
NIR-GECO2G provides reliable stimulation responses in wild-type mice with fluorescence imaging. (a) Widefield fluorescence expression in a wild-type mouse and ROIs selected for processing. (L = left, R = right, A = anterior, B = posterior). (b) Example detrended contralateral and ipsilateral fluorescence traces over 20 trials of left hindpaw electrical stimulation. Each red line represents the timing of a single 50 ms stimulus (thin red line) or a train of 10 ×20 ms stimuli (thick red line). (c) Δ*F*/*F* responses in wild-type mice (black line, 200 trials across 6 mice for single pulse stimulation and 70 trials across 3 mice for pulse train stimulation). Shading shows weighted standard error. Box charts show Δ*F/F*, time to peak, and decay time (red line = median, blue boxes = 25th and 75th quartiles, black whiskers = +/–2.7σ). (d) Δ*F*/*F* spatial trial-average responses for the left hindpaw stimulation experiment shown in (b) at different post-stimulation times. All scale bars = 1 mm.

For each mouse, a rigid registration transform was calculated based on the 10th frame of each set of reference, stimulation, or bleaching data to align all datasets across acquisitions and days. Once the transform was acquired for a dataset, it was applied to each frame in that dataset. Further rigid motion correction was applied to each stimulation session, using the originally registered frame as the reference. ∼1 mm diameter circular regions of interest (ROIs) were selected at the center of expression on the left and right hemispheres and traces were acquired over the experiment. Since the NIR fluorescence in any region includes contributions scattered from other expressing regions, a background ROI was selected on the skull outside of the area of expression to estimate this nonspecific background. For each experiment, background value was calculated by taking the mean value of this ROI over the entire experiment. Experiments were rejected if less than 50 % of their trials provided a response. Image stacks for each experiment were processed with a Kalman filter prior to analysis (gain = 0.4). For every trial, *F*_*0*_ was calculated as the mean value of the background-subtracted signal *F* over 6 frames (0.9 s) before stimulation. Δ*F/F* = $\frac{F–F_{0}}{F_{0}}$ was then calculated for all frames of each trial. Averages and standard errors were weighted based on the number of trials used per animal [26]. To determine peak and timing parameters of the Δ*F*/*F* response, the average -Δ*F*/*F* response of an animal’s experiments on an experimental day was fit to a gamma function $y=c*f_{\Gamma }(t,a,b)$ where $f_{\Gamma }$ =$\frac{t^{a-1}e^{-\frac{t}{b}}}{b^{a}\Gamma (a)}\;$, the probability distribution function of Γ(a)*.* For image results (Fig. 2D), gaussian smoothing was applied to F0 (σ = 12, kernel size 49x49 pixels).

### 2.6 FONT imaging and analysis

Fourteen days after injection, wild-type (n = 4) and *Blvra*^*-/-*^ (n = 4) mice were anesthetized under 1–1.5 % isoflurane (3 % isoflurane for induction) and their scalps were removed. A widefield fluorescence reference was taken with 660 nm LED illumination at 0.0025 mW/mm^2^. Ultrasound gel (Aquasonic 100 Clear, Parker Laboratories, USA) was placed on the heads of the mice, which were then coupled to the plastic membrane beneath the ultrasound transducer array. The OPO was pulsed at 20 Hz and switched between the eight wavelengths 670, 680, 690, 700, 735, 750, 800, and 850 nm (Fig. 1(a)), resulting in a final temporal resolution of 2.5 Hz. Laser output at the mouse head was ∼26 mJ at 680 nm over an area of ∼1.5 cm^2^, measured on a pyroelectric energy sensor (ES220C, Thorlabs, USA). Electrical stimulation was performed in the right hindpaw every 10 seconds for 20 trials. Afterwards, a second widefield fluorescence reference was taken with the same parameters as the initial reference.

After acquisition, back-projection reconstruction [27] was performed using a custom MATLAB script, resulting in volumetric images with 50 μm lateral and 100 μm axial image resolution for 1 cm in each dimension. Signals were filtered between 0.1 MHz and 3 MHz. Back-projection involved element interpolation to increase the number of transducers by a factor of 9 [28]. Prior to back-projection, data was deconvolved with an array impulse response determined by obtaining the OA signal of a 50 μm polyethylene sphere (Cospheric, USA) placed at the array focus [29]. Energy correction was applied based on average laser energy at each wavelength. To correct for potential light fluence differences at the brain between mice, a grayscale mask of blood vessels was used: the mean value of the mask across the first four wavelengths (for 6 first frames) was used to normalize the signal from each animal.

Spatial correction was performed for both lateral and depth dimensions. The lateral dimension is affected by both inhomogeneity in both light fluence and ultrasound array sensitivity, each resulting in lower relative signal towards the edges of the field-of-view. To address this, we determined a relationship between the fluorescence reference image and the maximal intensity projection of NIR-GECO2G distribution. This approximated a two-dimensional cosine, which was divided out from all voxels depending on lateral position (Supplementary Fig. S1). The depth dimension is affected by light attenuation in tissue, which can be modeled as exponential decay due to absorption and scattering. After defining a brain mask and determining the tissue surface, depth correction along the *z* dimension was applied for every lateral position [20]. Following reconstruction and corrections, a non-negative least-squares unmixing algorithm [30] was applied to each repetition of the wavelength sequence to separate oxyhemoglobin (HbO), deoxyhemoglobin (HbR), and NIR-GECO2G. HbO and HbR were then summed to obtain total hemoglobin (HbT), displayed in the image results as the blood component. For ROI analyses, ROIs of size 7 × 7 × 3 voxels (350 × 350 × 300 μm) were chosen, generally centered around centroids of maximal expression.

### 2.7 Multimodal fiberscope-based imaging and analysis

To explore the potential of simultaneous widefield fluorescence and FONT, we imaged three wild-type mice expressing NIR-GECO2G (5 weeks old, 2 weeks following injection) using the ultrasound array discussed in Section 2.1 with a fiberscope-based configuration (Zibra Corp, USA; Fig. 5(a)). The fiberscope had an optical imaging resolution of ∼56 μm (Supplementary Fig. S3). Wavelengths were sequentially switched between 680 nm, 805 nm, and 900 nm at 10 Hz, and pulse intensity was ∼0.07 mJ/mm^2^ per 680 nm pulse. The FONT data was captured by the ultrasound array while fluorescence data was captured by the sCMOS camera (Prime BSI, Teledyne Photometrics, USA) at 680 nm using a 775±70 nm bandpass emissions filter (FF01–775/140–25, Semrock, USA) between the imaging arm of the fiberscope and the camera. Spatial registration between the modalities was first performed approximately by scaling the horizontal maximal intensity projection of optoacoustic data and the widefield fluorescence reference, each with known pixel dimensions, to the same scale. The optoacoustic projection was then made partially transparent, overlaid and manually aligned to the fluorescence image based on aligning blood vessel outlines between the two modalities.

To visualize regions which display bleaching in FONT at 680 nm, a generalized linear model was fit using the MATLAB glmfit script. A predictor was determined by fitting a double exponential to the bleaching curve extracted from the fluorescence data. The signal from each FONT voxel at 680 nm was entered as a response to this predictor in the generalized linear model, resulting in t-scores which indicate the correlation of each voxel to the bleaching trend seen in fluorescence.

## 3 Results

### 3.1 NIR-GECO2G displays robust fluorescence responses to stimulation in vivo

First, we performed widefield fluorescence imaging of wild-type mice expressing NIR-GECO2G during electrical hindpaw stimulation to investigate the *in vivo* performance of NIR-GECO2G in mice. Hemispheric and background ROIs with diameters of ∼1 mm were chosen for each animal (Fig. 2(a)). Fig. 2(b) shows example time traces extracted from contralateral and ipsilateral ROI over an entire experiment with 20 trials, shown with bleaching de-trended, with the trend obtained by approximating a double exponential fit. After calculating Δ*F/F* for each trial, all trials were averaged and revealed consistent stimulus-locked fluorescence responses. Data used here includes trials from all experimental days, as Δ*F/F* remains stable despite progressive photobleaching (Supplementary Fig. S2.). The peak Δ*F/F* value across all wild-type animal experiments was −1.38 ± 0.13 % for a single stimulus, and −1.6 ± 0.18 % for multiple stimuli (Fig. 2(c)). These values are several-fold higher than the responses reported for NIR-GECO1 in similar conditions [10]. Time to peak was ∼0.67 s for single stimuli and ∼0.83 s for multiple stimuli, and decay time was ∼1.2 seconds for single stimuli and ∼1.4 s for multiple stimuli. Fig. 2(d) shows the spatial-temporal evolution of Δ*F/F* map for the experiment shown in Fig. 2(b).

### 3.2 Biliverdin reductase-A-deficient mice exhibit elevated fluorescence

**Fig. 3:**
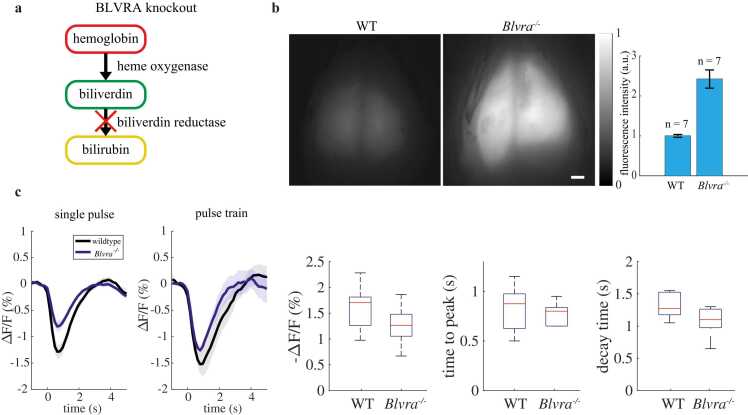
*Blvra*^*-/-*^ mice exhibit increased fluorescence signal over wild-type (WT) mice. (a) Diagram of heme catabolism, with the crossed-out enzyme depicting the result of the BLVRA knockout. (b) Widefield fluorescence images of representative wild-type and *Blvra*^*-/-*^ mice under the same illumination intensity. Scale bar = 1 mm. Inset: comparison of average fluorescence intensity in multiple wild-type and *Blvra*^*-/-*^ mice (n=7 for each group), scaled to mean wild-type mouse intensity. Error bars show standard deviation. (c) Δ*F/F* responses in *Bl*vra^-*/-*^ mice with wild-type responses from Fig. 1(c) for comparison (black). Shading areas show weighted standard error. Box charts show Δ*F/F*, time to peak, and decay time for wild-type and *Bl*vra^-*/-*^ mice under pulse train stimulation (red line = median, blue boxes = 25th and 75th quartiles, black whiskers = +/–2.7σ).

Next, we repeated the experiments in the previous section in *Blvra*^*-/-*^ mice in order to determine the baseline fluorescence strength and functional performance of NIR-GECO2G in the presence of heightened endogenous biliverdin. In *Blvra*^*-/-*^ mice (Fig. 3(a)), initial NIR-GECO2G fluorescence increased about 2.5-fold (Fig. 3(b)). Hindpaw stimulation responses were consistent and repeatable with an average of −0.85 ± 0.11 % Δ*F/F* for single stimuli and of −1.27 ± 0.10 % Δ*F/F* (Fig. 3(c)). This is less than the signal change for wild-type mice, potentially due to the increased overall fluorescence resulting in a greater proportion of background signal arriving from more non-responding regions. Time to peak was ∼0.83 s for single stimuli and ∼0.77 s for multiple stimuli, and decay time was ∼1 s for single stimulations and ∼1.2 seconds for multiple stimuli.

### 3.3 The OA signature of NIR-GECO2G is observable in vivo

**Fig. 4:**
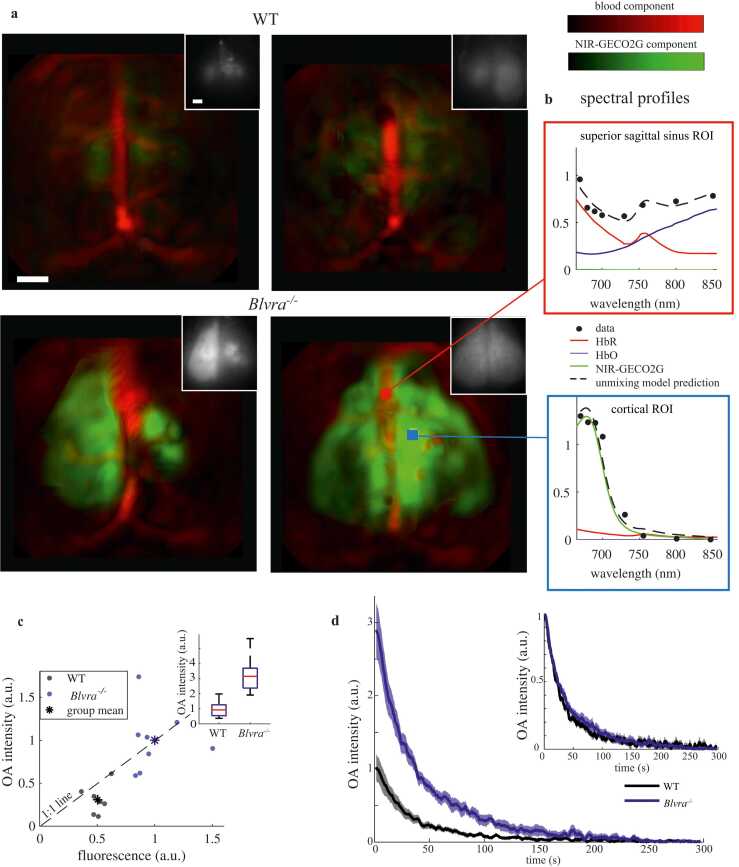
OA signature of NIR-GECO2G *in vivo*. (a) Maximal intensity projection of NIR-GECO2G distribution in representative wild-type (WT) and *Blvra*^*-/-*^ mice (red = blood component, green = NIR-GECO2G component), scaled by blood mask intensity (scale bars = 1 mm). In the righthand *Blvra*^*-/-*^ mouse, the red and blue ROIs are the source ROIs used in (b). Insets: widefield fluorescence reference images of each respective animal. (b) Spectra and unmixing model predictions for selected blood vessel (red) and cortical (blue) in (a). (c) OA signal vs fluorescence in wild-type (black, n = 4 mice, 7 total ROIs) and *Blvra*^*-/-*^ (blue, n = 4, 8 total ROIs) mice. The dashed line denotes x = y, and data are scaled to the mean value of results in *Blvra*^*-/-*^. Inset: OA signal comparison between wild-type and *Blvra*^*-/-*^ mice, scaled to mean value of results in wild-type mice. (p<0.001, Wilcoxon rank sum test) (d) Bleaching comparison of dye component in wild-type (black) mice and *Blvra*^*-/-*^ (blue) mice. Shaded area indicates standard errors of the mean. Inset: same data, with the maximum value of each bleaching curve normalized to unity.

Following widefield experiments, we attempted to image NIR-GECO2G using multispectral FONT. We performed 8-wavelength FONT imaging experiments as well as baseline/final widefield fluorescence experiments on both wild-type and *Blvra*^*-/-*^ animals to compare outcomes and to determine if the FONT signal differences correlate with differences we observed in fluorescence. After spectral unmixing, the distribution of NIR-GECO2G is clearly visible and resembles the distribution of fluorescence expression (Fig. 4(a)). Fig. 4(b) shows the model predictions of the component contributions to the example volumetric ROIs selected in the spectral signature in the cortical and superior sagittal sinus ROIs outlined in the lower right image in Fig. 4(a).

Across animals and hemispheres, we next examined the correlation of inter-ROI FONT-fluorescence values for ROIs placed at the centroid of expression in each hemisphere (Fig. 4(c), one wild-type animal only expressed in one hemisphere, and is only represented by one ROI). Here, the FONT signal was calculated as the difference of the NIR-GECO2G component between a mean difference of initial frames vs. final frames, where signal is bleached away; fluorescence is calculated similarly, with fluorescence values determined as the difference in image intensity between the widefield reference images taken pre- and post-FONT. Similarly to the gain in fluorescence, we found that *Blvra*^*-/-*^ animals (n = 4) have a factor of 2.7 greater signal in the NIR-GECO2G unmixed component compared to wild-type animals (n = 4), and the scatter of individual ROI values was highly correlated.

Photobleaching occurs rapidly under the illumination regime used in these experiments, with most of the NIR-GECO2G component diminished after 90 seconds (Fig. 4(d) and Supplementary Video 1). Normalizing the bleaching signal of wild-type and *Blvra*^*-/-*^ mice shows that both groups bleach at the same rate under these experimental conditions (Fig. 4(d) inset).

### 3.4 Multimodal imaging and photobleaching characterization

**Fig. 5:**
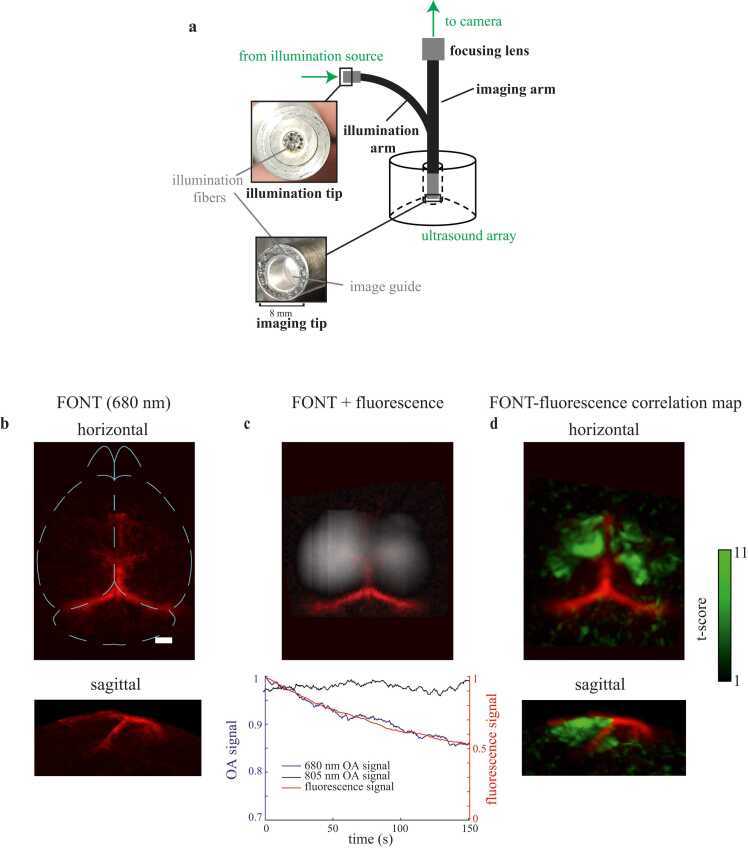
Multimodal NIR-GECO2G imaging and photobleaching. (a) Diagram of fiberscope imaging setup. (b) Maximal intensity projections of FONT volumes recorded at 680 nm. Scale bar = 1 mm. (c) Fluorescence signal (grayscale) overlaid on the horizontal maximal intensity projection of blood vessels in FONT from (b) (red). The plot shows signal over time in a FONT ROI (blue) and simultaneously in a corresponding fluorescence ROI (red) at 680 nm, as well as the FONT ROI at 805 nm (black). (d) 3D map of the FONT temporal trace fits to the fluorescence bleaching trend (t-score maximum intensity projections in green superimposed on red FONT blood vessel projections from (b)).

We next used the fiberscope configuration to gather simultaneous fluorescence and multi-spectral FONT information (at 680, 805, and 900 nm), during hindpaw stimulation. Blood vessels are visible in the FONT images taken at the 680 nm excitation wavelength, here the superior sagittal sinus and the transverse sinuses (Fig. 5(b)). Fluorescence is concurrently visible through the fiberscope at 680 nm. While an OA signature of functional response is not readily identifiable at this single wavelength and at lower illumination energy used in this particular experiment, a decreasing OA signal at 680 nm is recovered in a volumetric ROI corresponding to a bleaching trend the fluorescence signal in a corresponding ROI (Fig. 5(c)). In the 805 nm frames, no bleaching is observed for the same ROI, as expected due to the lack of NIR-GECO2G absorption at 805 nm. While the OA signal seems to bleach less as a percentage of initial signal, this is at least partially due to the presence of other absorbing components of tissue generating OA signal. This is in contrast to fluorescence signal, which has a much lower background signal.

Finally, we determined the t-scores of FONT voxel correlations with this photobleaching fluorescence signal using a generalized linear model (see Section 2.6 for details), and found that their volumetric statistical map appears to be distributed in cortex in a similar pattern to fluorescent expression (Fig. 5(d)). This suggests that this is a viable method for observing protein distribution, potentially using only a single wavelength.

## 4 Discussion

In this study we have used multimodal FONT and fluorescence imaging to explore the *in vivo* characteristics of the current state-of the-art NIR GECI, NIR-GECO2G. Our results showed that NIR-GECO2G is compatible with *in vivo* functional widefield imaging experiments over multiple days with high SNR (Fig. 2 and Supplementary Fig. S2). We also visualized NIR-GECO2G expression *in vivo* with FONT, the first time this has been reported with an NIR GECI (Fig. 4). Using fluorescence together with FONT, we validated that the signature seen in FONT images is the expected NIR-GECO2G signal seen in fluorescence.

The key advance highlighted in this study is the ability to image an NIR GECI using FONT and multimodal imaging. Earlier FONT work relied on visible GCaMPs excited at 488 nm, which provided limited depth of imaging in the mouse brain [4], while complementary work using NIR wavelengths ≥650 nm with other types of indicators achieved deep OA imaging, e.g., amyloid-β imaging throughout the mouse brain [31]. Here, we explored the next step, namely, using a NIR GECI for functional imaging; transcranial NIR imaging with our system can provide >5 mm imaging depth in the mouse brain (Fig. 1(d)). Further, we used fluorescence imaging together with FONT to provide complementary information and to validate initial FONT results. Widefield fluorescence images in the NIR suffer from increased nonspecific background due to tissue light scattering while FONT does not, and therefore can localize signal source more accurately. Conversely, FONT imaging in protein-labeled tissue suffers from high vascular background signals, leading to possible misidentification of functional signals, while this is not a significant concern for fluorescence imaging. Furthermore, we also used fluorescence information to improve the lateral spatial homogeneity of the FONT signal, and to corroborate signal distribution and bleaching patterns seen in FONT. Multimodal imaging will be a crucial component of future advances and applications, in some cases for implementing multimodal indicators as in this study while in other cases gathering complementary information in each modality, thus leading to multiplexing of diverse contrasts; for example, vascular or hemodynamics signal obtained optoacoustically can be combined with fluorescence for molecular labeling or functional information [15], [20], [32], [33].

In addition, we developed and evaluated methods to increase NIR-GECO2G image quality in FONT. For example, we found that *Blvra*^*-/-*^ mice provided greater than twofold increased NIR-GECO2G signal in the fluorescence and FONT imaging modes. To our knowledge, this is the first use of *Blvra*^*-/-*^ mice for *in vivo* functional Ca^2+^ brain imaging. These findings are consistent with prior findings showing increased signal strength of biliverdin-based proteins in mouse embryonic fibroblasts [18] and *in vivo* in mouse liver [34]. Our results also suggest that the *Blvra*^*-/-*^ mouse line provides a way to enhance and prolong the signal of biliverdin-based fluorescent proteins in future *in vivo* OA imaging studies. Interestingly, while NIR-GECO2G baseline fluorescence was increased in *Blvra*^*-/-*^ mice, functional responses were similar but slightly lowered. This is potentially due to increased nonspecific scattered background light, and it is worthwhile to note that these responses were still larger than NIR-GECO1 responses in wild-type mice. While *Blvra*^*-/-*^ mice present a powerful method to boost NIR fluorescent protein and NIR GECI expression *in vivo*, BLVRA deficiency may pose potential concerns for neuroscience studies due to elevated levels of oxidative stress [35], [36] and the influence of BLVRA in insulin signaling cascades leading to implication in neurodegenerative conditions such as Alzheimer’s Disease [37], [38]. Despite these possible issues, the *Blvra*^*-/-*^ line was reported to have regular indicators of health, including reproduction, weight, and lifespan [18].

One limitation of NIR-GECOs is their rapid photobleaching [22], which was observed in both our fluorescence and FONT experiments. However, we find that this effect is light-dose dependent (Supplementary Fig. S4). Pulse energies were high to maximize SNR in these initial characterization experiments, so the observed bleaching can be partially mitigated by using lower light intensities in the future [39], similarly to our approach in Section 3.4. This may work despite reduced intensities also reducing image SNR, because our primary limiting factors for functional imaging appear to be different: pulse-to-pulse illumination wavefront fluctuations and photobleaching. Reducing photobleaching in experiments should allow us to perform longer experiments, capturing more trials to average over while maintaining sufficient signal quality. Fortunately, improved photostability is also one of primary optimization targets being pursued in the development of next-generation NIR Ca^2+^ indicators. Photostability can possibly be improved by developing an indicator with a positive fluorescence response to Ca^2+^ instead of a negative response manifested by NIR-GECOs. Beyond photostability, the sensitivity, speed, absorption cross-section, and biliverdin dependence can all be optimized, as has been successful with the GCaMP family of indicators[10], which have improved multifold in all of these parameters in the decade and a half since early GECI GCaMP3 was released and demonstrated for *in vivo* use [40]. jGCAMP8, for example, was evolved until a ∼2 ms half-rise time was achieved, almost tenfold faster than prior GCaMP variants [10]. NIR GECI developers are working to improve these probes by implementing similar methods as ones that were used to improve GCaMP, such as structure-guided mutagenesis and directed evolution targeted to components such as peptides or protein-Ca^2+^ domain interfaces. Efforts to improve NIR GECIs have already led to a new, brighter generation of NIR-GECO, the NIR-GECO3 series, as well as to an effective new method to discover more future single fluorescent protein-based biosensors [14].

Development of OA-specific functional probes is a major need and challenge for the future of FONT. For deep-tissue applications, it is crucial that indicator parameters be specifically optimized for OA signal generation, as optimization to fluorescence-relevant quantities like quantum yield may have undesired effects on OA emission. One exciting possibility for optimization of NIR indicators for OA is the recent developments of chemigenetic indicators, such as HaloCaMP [41] and WHaloCaMP [42]. These consist of a protein with a Ca^2+^ sensor domain that modulates the fluorescence of a separately delivered dye ligand. Screening and developing FONT-compatible chemigenetic platforms may provide a potential path forward for functional indicators as optimizations for absorption (based on chemical synthesis of dyes) and Ca^2+^ sensing (protein engineering) can be addressed separately. Thus far, efforts towards FONT-optimized chemigenetic Ca^2+^ reporters have resulted in ‘acoustogenic’ probes which outperform NIR-GECO1 in FONT-based Ca^2+^ sensing *in vitro* [43].

With its mesoscale resolution and high temporal resolution across much of the mouse brain, FONT is a promising tool for directly probing neural activity in distributed brain systems which exhibit activity throughout the brain. Our results further suggest that multimodal FONT-fluorescence dynamic imaging may offer further complementary advantages towards characterizing and optimizing molecular probes that can help realize this potential. By demonstrating the ability to detect state-of-the-art NIR sensors like NIR-GECO2G *in vivo*, we have achieved an essential step towards this goal.

## 5 Conclusions

We have demonstrated the feasibility of NIR-GECO2G for multi-day imaging experiments *in vivo* in mice. Additionally, we have used *Blvra*^*-/-*^ mice to successfully increase NIR-GECO2G signal in the fluorescence and FONT imaging modes. Most significantly, this work includes the first FONT images of a NIR Ca^2+^ indicator *in vivo*. With future developments in NIR functional probes, we anticipate that FONT can become a powerful tool for fast mesoscale *in vivo* imaging of Ca^2+^ activity across the entire mouse brain.

## Funding sources

This work was supported by the National Institute of Neurological Disorders and Stroke of the National Institutes of Health under award number RF1NS126102 and also supported in part by a grant from Research to Prevent Blindness to NYU Langone Health's Department of Ophthalmology. Work by R.E.C. and Y.Q. at the University of Alberta was supported by the Canadian Institutes of Health Research (FS-154310) and the 10.13039/501100000038Natural Sciences and Engineering Research Council of Canada (RGPIN-2018–04364).

## CRediT authorship contribution statement

**Sarah F Shaykevich:** Writing – review & editing, Writing – original draft, Visualization, Software, Investigation, Formal analysis. **Justin P Little:** Writing – review & editing, Writing – original draft, Supervision. **Yong Qian:** Writing – review & editing, Resources. **Marie-Eve Paquet:** Writing – review & editing, Resources. **Robert E Campbell:** Writing – review & editing, Resources, Funding acquisition, Conceptualization. **Daniel Razansky:** Writing – review & editing, Software, Funding acquisition, Conceptualization. **Shy Shoham:** Writing – review & editing, Writing – original draft, Supervision, Funding acquisition, Conceptualization.

## Declaration of Competing Interest

The authors declare that they have no known competing financial interests or personal relationships that could have appeared to influence the work reported in this paper.

## Data Availability

Data will be made available on request.

## Appendix A Supplementary material

Supplementary material

**Video 1** Maximal intensity projections of unmixed FONT volumes of a *Blvra*^*-/-*^ mouse over time. Red = sum of HbR and HbO components, green = NIR-GECO2G component.
