# Supplementary material for: Multimodal fluorescence-optoacoustic in vivo imaging of the near-infrared calcium ion indicator NIR-GECO2G
Source: Photoacoustics. 2024 Nov 28;41:100671. doi: 10.1016/j.pacs.2024.100671 (PMC11732225; doi:10.1016/j.pacs.2024.100671)
Supplement: Supplementary file 1 — Supplementary material [file mmc1.docx]

**Figure S1.** Lateral spatial multimodal correction of FONT images.
 The NIR-GECO2G fluorescence expression image captured by widefield fluorescence imaging (a) contains more signal diffusion than the matching optoacoustic MIP (b), but is more evenly illuminated and imaged. The OA image in (b) contains non-diffuse spatial information about NIR-GECO2G expression, but suffers from spatial inhomogeneities due to illumination and array sensitivity. (c) Spatially corrected MIP of the data in (b). Inset: correction factor which each voxel is divided by based on XY position. The cosine correction factor is defined at each (x,y) coordinate position by $\left( \frac{\cos\left( \frac{2\pi x}{x_{0}} \right)+1}{2}\cdot\frac{\cos\left( \frac{2\pi y}{y_{0}} \right)+1}{2} \right)^{2}$, where *x_0_* = *y_0_* = 13.3 mm, was determined by comparing factors between profiles of fluorescence images and profiles of NIR-GECO2G expression in OA MIPs. This factor was windowed to avoid noise amplification at the edges and corners of the image. All scale bars = 1mm.

**Figure S2.** Fluorescence intensity loss and Δ*F/F* stability throughout multiday experiments.
(a) Overall fluorescence decreases over several days of experiments. The first scatterplot point for each day is the initial fluorescence, and the following points are taken after each of three 16-minute bleaching session (except for day 10, when the second reference point is taken after stimulation experiments). Error bars show standard error of the mean. The first experimental day is 14 days after injection. (b) While fluorescence decreases over time, functional responses remain fairly stable. Scatter points show mean Δ*F/F* on each experimental day. Dashed lines show the mean of all points, and shading shows average S.E.M. across all days. Each plot includes wild-type (black, n = 3) and *Blvra^-/-^* (blue, n = 3) mice.

| 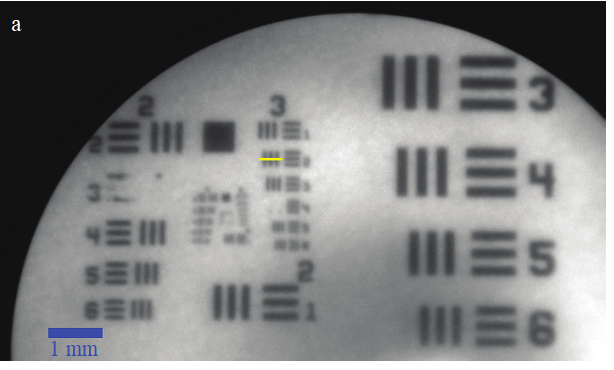 |
| --- |

**Figure S3.** Fiberscope widefield imaging characterization. (a) Fiberscope image of USAF 1951 target through water. The yellow line shows the location for the intensity profile in (b).

**Figure S4.** Dose dependency of photobleaching.
(a) Comparison of bleaching rates with constant light doses. NIR-GECO2G-expressing wild-type mice were illuminated for 210 s in cycles of 200 ms at either a high intensity 9.2 mW/mm^2^ and 8% duty cycle (blue, n=2), medium intensity 3.8 mW/mm^2^ and 20% duty cycle (green, n=1) or lower intensity 1.25 mW/mm^2^ and 50% duty cycle (red, n=1). All parameter combinations resulted in an average intensity of ~0.035 mW/mm^2^, and traces follow a similar trend (each normalized between initial and final fluorescence values).

(b) Comparison of photobleaching under different illumination conditions. NIR-GECO2G-expressing wild-type mice were illuminated with either 0.3 mW/mm^2^ of continuous 660 nm LED light (black, n = 2) or 0.1 mJ/mm^2^ 680 nm nanosecond laser pulses (blue, n = 3) directed through a fiber bundle at 20 Hz. To compare photobleaching in terms of light absorbed, we convert each illumination measurement to cumulative light dose (mJ/mm^2^) by multiplying LED illumination intensity by exposure time or multiplying laser pulse energy per area by number of pulses. While bleaching occurs more rapidly under high-energy laser pulses, the scale of bleaching is more comparable when quantified with respect to total light dose.
